# Decellularized skeletal muscles display neurotrophic effects in three‐dimensional organotypic cultures

**DOI:** 10.1002/sctm.20-0090

**Published:** 2020-06-24

**Authors:** Paolo Raffa, Valentina Scattolini, Mattia Francesco Maria Gerli, Silvia Perin, Meihua Cui, Paolo De Coppi, Nicola Elvassore, Paola Caccin, Camilla Luni, Anna Urciuolo

**Affiliations:** ^1^ Veneto Institute of Molecular Medicine Padova Italy; ^2^ Women's and Children's Health Department University of Padova Padova Italy; ^3^ University College London Great Ormond Street Institute of Child Health London UK; ^4^ Shanghai Institute for Advanced Immunochemical Studies (SIAIS) ShanghaiTech University Shanghai People's Republic of China; ^5^ Industrial Engineering Department University of Padova Padova Italy; ^6^ Biomedical Science Department University of Padova Padova Italy; ^7^ Institute of Pediatric Research (IRP), Fondazione Città della Speranza Padova Italy

**Keywords:** 3D culture, axons, decellularized muscle, ECM, innervation, neurons, organotypic culture, spinal cord

## Abstract

Skeletal muscle decellularization allows the generation of natural scaffolds that retain the extracellular matrix (ECM) mechanical integrity, biological activity, and three‐dimensional (3D) architecture of the native tissue. Recent reports showed that in vivo implantation of decellularized muscles supports muscle regeneration in volumetric muscle loss models, including nervous system and neuromuscular junctional homing. Since the nervous system plays pivotal roles during skeletal muscle regeneration and in tissue homeostasis, support of reinnervation is a crucial aspect to be considered. However, the effect of decellularized muscles on reinnervation and on neuronal axon growth has been poorly investigated. Here, we characterized residual protein composition of decellularized muscles by mass spectrometry and we show that scaffolds preserve structural proteins of the ECM of both skeletal muscle and peripheral nervous system. To investigate whether decellularized scaffolds could per se attract neural axons, organotypic sections of spinal cord were cultured three dimensionally in vitro, in presence or in absence of decellularized muscles. We found that neural axons extended from the spinal cord are attracted by the decellularized muscles and penetrate inside the scaffolds upon 3D coculture. These results demonstrate that decellularized scaffolds possess intrinsic neurotrophic properties, supporting their potential use for the treatment of clinical cases where extensive functional regeneration of the muscle is required.

## Graphical abstract

Decellularized muscles show direct neurotrophic properties. Here, we developed a three‐dimensional in vitro model in which organotypic sections of fetal spinal cord were cultured in presence or in absence of decellularized muscles. Decellularized muscles, which proteomic composition includes both myofibers and peripheral nerve components, sustained axon sprouting and attraction of neurons.

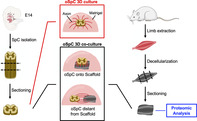

**Significance statement**

Skeletal muscle decellularization allows the generation of natural scaffolds that retain the extracellular matrix mechanical integrity, biological activity, and three‐dimensional (3D) architecture of the native tissue. State‐of‐the‐art studies report the evidence of a pro‐innervation ability of decellularized muscles when implanted in in vivo models. It was found that decellularized muscles preserve extracellaral matrix proteins of both muscular and peripheral nervous systems. To investigate whether decellularized scaffolds could per se attract neural axons, organotypic sections of spinal cord were cultured three dimensionally in vitro, in the presence or absence of decellularized muscles. This study found that neural axons extended from the spinal cord are attracted by the decellularized muscles and penetrate inside the scaffolds upon 3D coculture. These results demonstrate that decellularized scaffolds possess intrinsic neurotrophic properties, supporting their potential use for the treatment of clinical cases where extensive functional regeneration of the muscle is required.

## 1 INTRODUCTION

Tissue engineering combines extracellular natural and/or synthetic scaffolds (biomaterials) with stem cells and growth factors for the development of regenerative medicine strategies and the treatment of diseased tissues.^1^ Synthetic scaffolds have the advantage over natural biomaterials in that their structure, topography, and mechanical properties can be finely tuned to design an optimal environment for a particular biological application.^1^ Despite incredible improvements have been achieved in biomaterial manufacturing, many challenges remain in preparing scaffolds that recapitulate in vitro, the complexity of the tissue microenvironment. The peculiar combination of the tissue‐specific extracellular matrix (ECM) biochemistry, biomechanics, and three‐dimensional (3D) organization cannot be fully reproduced in the lab.^2^, ^3^, ^4^ Therefore, there has been increasing interest in using naturally derived ECM itself, as decellularized (decell) tissues or whole organs, where such complexity can instead be preserved.^5^, ^6^, ^7^

The decellularization process removes cellular and nuclear content of the native tissue, while retaining ECM mechanical integrity, biological activity, and 3D architecture.^6^ Decell scaffolds are highly biocompatible and show absence of rejection after allogeneic or xenogeneic transplantation.^8^ Altogether, these properties make them an important and promising alternative biomaterial for the treatment of clinical cases as traumatic injuries, surgical ablations, and congenital malformations.^9^, ^10^ Indeed they have already been obtained from different organs and used for regenerative medicine strategies in animal models, as well as in clinical trials.^9^, ^10^

In particular, decell muscles have been shown to promote muscle regeneration in volumetric muscle loss models.^7^ We recently developed perfusion methods for the generation of skeletal muscle scaffolds which retain 3D structural organization of the tissue, as well as ECM components and growth factors. Decell muscles were used as xenograft to promote tissue regeneration in a murine model of volumetric muscle loss, which also allowed innervation and regeneration of the neuromuscular junctions.^11^ In agreement with this, other studies have demonstrated that, when implanted in vivo, decell muscles are not only able to restore muscle mass, but also trigger the regeneration of the nervous system with overall functional recovery.^12^, ^13^ Finally, it was recently reported that decell muscles guide nerve regrowth in a diaphragmatic hernia mouse model.^14^

During in vivo peripheral nerve regeneration, injured axons are able to elongate into the distal nerve stump if they find a permissive substrate. This is mainly provided by trophic support from Schwann cells, connective cells, and ECM. Eventually, regenerating axons will be mostly able to reach the distal target organs and reinnervate them, thus allowing for the recovery of lost functions.^15^, ^16^ The ECM is an essential player required for the formation of axonal tracts as well as for the maturation and function of synapses in the peripheral nervous system.^16^, ^17^, ^18^, ^19^, ^20^ As extensively demonstrated by in vitro studies and tissue engineering strategies, axonal regrowth and guidance are enhanced by ECM components, including collagen IV or laminin.^16^, ^17^, ^18^, ^19^, ^20^ In agreement with this, decell scaffolds prepared from nervous system‐derived tissues (including decell nerves) have been proved to support long‐distance axon regeneration in peripheral nerve injury in animal models^21^, ^22^, ^23^, ^24^ as well as in patients.^25^, ^26^, ^27^

Other decell tissues used for nerve repair in in vivo models include scaffolds derived from small intestinal submucosa,^28^ amniotic tissue grafts,^29^, ^30^ and umbilical cord.^31^ Few studies also reported the ability of implanted decell muscles to repair nerve injury in vivo^27^, ^32^, ^33^, ^34^, ^35^.

The evidence that decell muscles promote myogenesis have been observed both in in vivo and in vitro models, indicating that these scaffolds directly preserve biological activity able to guide myogenic cells toward the generation of myofibers.^12^, ^36^ On the contrary, innervation of decell muscles has only been observed in in vivo models.^11^, ^12^, ^14^ Therefore, it remains unclear whether the neurotrophic properties of the scaffolds observed in vivo could be the result of the overall regenerative response to the implant, or if decell muscle could directly promote axon invasion. The implementation of the nervous system is essential for skeletal muscle tissue functionality, which is a necessary feature for the future use of decell muscles in clinical application and 3D in vitro modeling. The purpose of this study was, therefore, to investigate the direct ability of decell scaffolds to promote axonal sprouting and invasion in vitro. To do so, we developed a 3D coculture system of organotypic spinal cord slides (oSpC) and decell muscles. This experimental approach allows the study of the neurotrophic effect of the scaffolds by excluding the influence of other cellular and/or systemic components that instead exist in vivo.

## 2 MATERIALS AND METHODS

### 2.1 Animals

For decellularized muscle preparation, 250 to 350 g male or female Sprague Dawley rats were used; all the procedures performed on animals were in accordance with Animals (Scientific Procedures) Act 1986. For spinal cord cultures, E14 fetuses were derived from pregnant Sprague Dawley rats purchased from Charles River Laboratories; all the procedures performed on animals were in accordance with Italian National laws and policies (D.L. n. 26, 14 March 2014), with the guidelines established by the European Community Council Directive (2010/63/EU), and were approved by the Italian Ministry of Health (authorization number: 81/2017 PR).

### 2.2 Decellularized muscle preparation

Rats (250‐350 g) were used as a source of muscle for decellularization. The leg was dissected from the rest of the body by splitting the pelvis at the pubic symphysis and the sacroiliac joint. We performed decellularization as previously described.^11^ Briefly, a 24 G cannula was inserted into the abdominal iliac artery and advanced distally to allow perfusion condition at a flow rate of 1 mL/min. Limbs were perfused with 0.25% SDS (Sigma) for 72 hours and washed in deionized water for 48 hours. After decellularization, the muscles of interest were dissected, treated with 137Cesium irradiator (IBL 437C), and preserved at 4°C, in phosphate buffered saline (PBS, Gibco) with 1% Penicillin/Streptomycin (P/S, Gibco).

### 2.3 Proteomic sample preprocessing

The decellularized matrix was freeze‐dried for 72 hours (Labconco FreeZone Triad Freeze Dry Systems), milled into a thin powder using a mini‐mill (Thomas Wiley, mesh 40), and then lyophilized. The lyophilized decell powder batches, derived from three decellularization processes, were resuspended in 4% SDS (Sigma‐Aldrich). Protein extraction was performed by heating at 90°C for 10 minutes, and centrifuging at maximum velocity for 10 minutes at 4°C. Extracted proteins were reduced in 0.1 M dithiothreitol (DTT) at 95°C for 5 minutes, dissolved in 8 M urea solution after cooling down to room temperature (RT), alkylated with 55 mM iodoacetamide for 30 minutes at 25°C in the dark. Alkylated proteins were purified using Microcon YM‐10 filter unit (MRCPRT010, Millipore) eight times at 14 000*g* for 40 minutes followed by trypsin (Promega) digestion for 16 hours at 37°C. Digested peptides were eluted with 100 mM TEAB buffer three times, followed by desalting and vacuum drying. One hundred micrograms of peptides were labeled by 6‐plex Tandem Mass Tag (TMT, Thermo Fisher Scientific) according to manufacturer's instructions and resuspended in 30 μL 0.1% acetic acid for the following mass spectrometry analysis.

### 2.4 Proteomic liquid chromatography‐tandem mass spectrometry analysis

Protein identification by liquid chromatography tandem mass spectrometry (LC‐MS/MS) was performed using Thermo Fusion Mass Spectrometer with Thermo Easy‐nLC1000 Liquid Chromatography. Ninety minutes of LC‐MS gradients were generated by mixing buffer A (0.1% formic acid in water) with buffer B (0.1% formic acid in 80% acetonitrile (ACN) in water) by different proportions. Using nanospray ionization (NSI) as the ion source and Orbitrap as the detector, the mass scan Rang was at 300 to 1800 m/z, and the resolution was set to 120 K. The MS/MS was isolated by Quadrupole and detected by Ion trap, whose resolution was set to 60 K. The activation type was higher‐energy collisional dissociation (HCD).

### 2.5 Proteomic bioinformatics analysis

Peak list files were searched against UniProt *Mus musculus* reference proteome by Thermo Proteome Discoverer 2.2, due to the high similarity but more complete annotation of this proteome respect to that of *Rattus norvegicus* (Figure S1). Searches were performed using a 10 ppm precursor ion tolerance for total protein level profiling. The product ion tolerance was set to 0.02 Da in mascot TMT6 quantification searches. TMT6 modification (229.163 Da) and carbamidomethyl on cysteine (+57.021 Da) were set as static modifications. The oxidation of methionine residues (+15.995 Da) was set as a variable modification. Peptide‐spectrum matches were adjusted to a 1% and then assembled further to a final protein‐level false discovery rate of 1%. Proteins not identified in all three replicates or identified with a *q*‐value >0.05 were filtered out. Protein localization was annotated according to the following gene sets: matrisome (structural and associated),^37^ extracellular vesicle (GO‐CC:1903561), membrane (GO‐CC:0016020), cytoskeleton (GO‐CC:0005856), and mitochondrion (GO‐CC:0005739). Protein classification by tissue was performed by comparison with data from ProteomicsDB (a mass spectrometry‐based proteomic annotation of human tissues)^38^ for all tissues a part for skeletal muscle (not present) whose protein composition was obtained from the results of the Human Skeletal Muscle Proteome Project.^39^ ECM structural proteins from this study were searched in String database^40^ for their known interactions according to experimental evidence or curated databases. The resulting network was exported and graphically plotted in Cytoscape v. 3.7.1.

### 2.6 Organotypic spinal cord 3D culture

Isolated SpC were cut in three sections (apical, central, and caudal; ~1 × 1 × 2 mm) and cultured within 5 μL drop of 100% Matrigel Growth Factor Reduced (Corning 354 230) casted onto glass coverslip. For oSpC 3D culture onto decell muscles, scaffolds were cut in ~1 × 2 × 4 mm sections and put on a glass coverslip; then a single oSpC section was added onto each scaffold section and covered by 10 μL Matrigel droplet. To evaluate neural attractant effects of decell muscles, SpC sections were placed side by side with scaffold or with a sterile hydrophilic cotton gauze (~1 × 1 × 2 mm) onto a glass coverslip; 15 μL Matrigel droplet was used to embed the samples. The inert and decell scaffolds were placed approximately the same distance from each other (~1.5 mm) using a millimeter grid that was located under the petri dish at the moment of seeding. Samples were cultured in Neurobasal medium (Gibco 21 103 049), B‐27 supplement (Gibco 17 504 044) 1X, 2% Horse serum (Gibco 16 050 122), 0.5 mM GlutaMAX Supplement (Gibco 35 050 038), 25 μM 2‐Mercaptoethanol (Gibco 31 350 010), 25 μM L‐Glutamic acid (Sigma G5889), Gentamicin/Amphotericin (Gibco R01510), 10 ng/mL ciliary neurotrophic factor (CNTF, PeproTech 450‐13), and 10 ng/mL glial‐cell‐line‐derived neurotrophic factor (GDNF, Peprotech 450‐10). oSpC sections were maintained in culture for 14 days. Half medium was changed every 4 days.

### 2.7 Cell viability assay

Viability assay was performed using Calcein, AM (LifeTechnologies, C3099). Samples were washed twice in PBS and incubated for 30 minutes with 3 μM Calcein, AM (LifeTechnologies, C3099) in medium without serum. After Calcein staining, samples were washed twice in PBS and analyzed under fluorescence stereomicroscope Leica MZ16F and/or two‐photon microscope (Thorlabs or Scientifica).

### 2.8 Immunofluorescence

Samples were fixed in 3% paraformaldehyde for 45 minutes, washed twice in PBS, and analyzed in whole‐mount or in 20 μm longitudinal and cross‐sections. Samples were blocked and permeabilized in blocking solution, 0.5% Triton (Sigma) and 1% bovine serum albumin (Gibco) in PBS, for 2 hours at room temperature. Samples were incubated with primary antibody for 24 hours (whole‐mount) or overnight (sections) at 4°C, washed in PBS, and incubated with secondary antibodies for 2 hours at room temperature. The following primary antibodies resuspended in blocking solution were used: Rat anti‐α‐Laminin (Sigma, L0663) 1:100; Rabbit anti‐Laminin (Sigma, L9393) 1:100; Mouse anti‐βIII‐Tubulin (Tuj1—Biolegend, 801 202) 1:5000; Rabbit anti‐βIII‐Tubulin (Tuj1—SYSY, 302302) 1:500; Goat anti‐GFAP (Abcam, ab53554) 1:1000; Goat anti‐ChAT (Millipore, AB144P) 1:50; Rabbit anti NeuN (Abcam, ab104225) 1:500; Mouse anti‐Neurofilament H (NF‐H, Biolegend, 801 602) 1:200; Rabbit anti‐S100 beta (Abcam, ab52642) 1:100; Mouse anti‐Nkx6.1 (DSHB, F55A10) 1:25; Mouse anti‐ISL1/2 (DSHB, 39.4D5) 1:25. The following secondary antibodies diluted in blocking solution were used: Donkey anti‐mouse 488 (ThermoFisher, A21202) 1:200; Donkey anti‐rabbit 488 (ThermoFisher, A21206) 1:200; Donkey anti‐mouse 594 (ThermoFisher, A21203) 1:200; Donkey anti‐rabbit 594 (ThermoFisher, A31573) 1:200); Goat anti‐Rat Cy2 (Jackson, 112‐225‐167) 1:100; Donkey anti‐goat Cy3 (Jackson, 705‐165‐147). An amount of 10 μg/mL Hoechst 33342 (ThermoFisher, H1399) was used to stain nuclei.

### 2.9 Imaging acquisition and analysis

Samples were analyzed with the following microscopes: epi‐fluorescence Olympus BX60; fluorescence stereomicroscope Leica MZ16F, equipped with Canon EOS1000D camera; confocal Leica TCS SP5 microscope; confocal ZEISS LSM 800 microscope; wide‐field motorized stage Leica DM6B; modular multiphoton microscope (Bergano‐II, Thorlabs) coupled with two synchronized pulsed laser beams (excitation 800 nm). ImageJ software was used for image processing, contrast and intensity level adjustment, and 3D reconstruction. For directionality analysis, *Directionality* ImageJ plugin was used to analyze bright field images of the region between the oSpC and the scaffolds or the axons sprouting from the oSpC in absence of scaffolds. The plugin measured the amount of structures in a given direction every 2° (from −90° to +90°), where scaffolds were placed at 0° axis. Images with completely isotropic content are expected to give a flat histogram, whereas images in which there is a preferred orientation are expected to give a histogram with a peak at that orientation. The quantification was expressed as the mean of 4 to 6 independent biological replicates.

### 2.10 Statistical analysis

All the analyses were performed by using GraphPad Prism 6 software. Plotted data were expressed as mean ± SEM. We determined statistical significance by unequal variance Student's *t* test or one‐way ANOVA (analysis of variance) and Tukey's multiple comparison test or Kruskal‐Wallis and Dunn's multiple comparison test. A *P* value of less than .05 was considered statistically significant.

## 3 RESULTS

**FIGURE 1:**
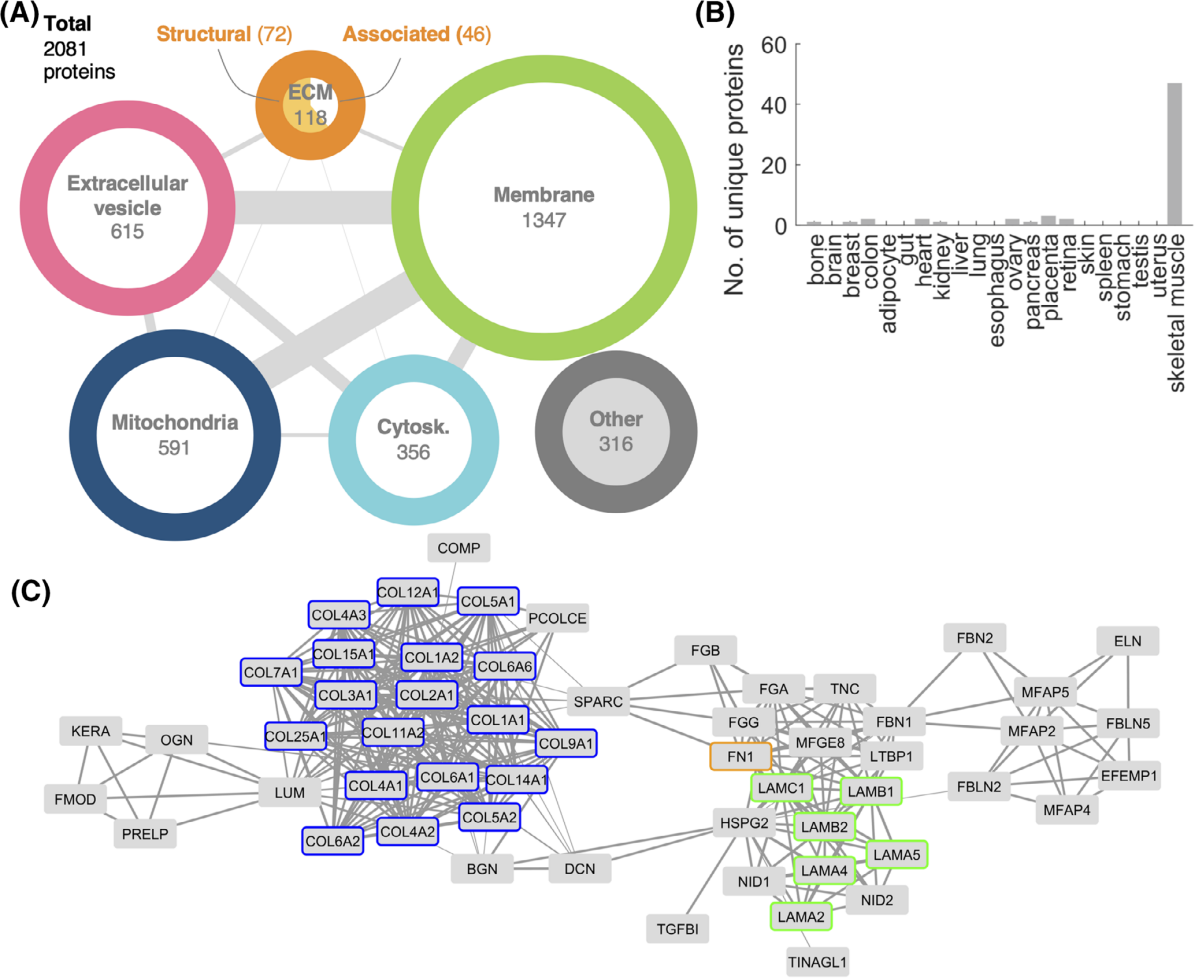
Proteomic analysis of decellularized skeletal muscle. A, Classification of the 2081 identified proteins based on their localization. Edge thickness is proportional to the number of proteins in common between the two linked categories; circle radius is proportional to the number of proteins in that category. B, Number of proteins identified in our data that are annotated exclusively to the indicated tissue. Reference data of human proteins per tissue are described in Section 2. C, Protein‐protein interaction network, including only ECM structural proteins having at least one reported neighbor. Collagens, laminins, and fibronectin are highlighted in blue, green, and orange, respectively. Edge thickness is proportional to confidence of interaction. ECM, extracellular matrix

Based on the cell instructive cue exerted by decell muscles upon implantation in volumetric muscle loss models^11^ and on the role of ECM during innervation,^16^, ^17^, ^18^, ^19^, ^20^ we first characterized the residual protein composition of decell scaffolds by mass spectrometry. After applying stringent filtering criteria, we identified 2081 proteins (Table S1). At the protein level, our results indicate that decell muscles are not only composed by structural ECM proteins, but these includes also numerous other associated proteins (Figure 1A). In detail, we identified 72 ECM structural proteins (including collagens, laminins, fibronectin, nidogen‐1 and ‐2, and proteoglycans) and 46 ECM‐associated proteins (Figure 1A and Table S2). Within the latter category, multiple proteases and other ECM remodeling enzymes were included. Among these, we identified cathepsins, a disintegrin, ADAM family metalloproteases (Adam10 and Adamtsl4), and protease inhibitors (as Serpineb1a, Serpineb6, Serpine2, Serpinf1, Serpinh1). We already demonstrated that decell muscles preserve single anucleated myofibers (that could also be isolated) and sarcolemmal proteins such as dystroglycans.^11^ Here, we confirmed these findings, identifying cytoskeleton proteins (including myosins, actins, and desmin) and sarcolemma proteins (GO‐CC: 0042383), including dysferlin (Dysf) and aquaporins (Aqp1, Aqp4 and Dag1) with its interacting partners (such as Lama2, Dmd, and Cav3; Figure 1A and Table S1). Direct associations between mitochondria and the cytoskeleton exist in myofibers.^41^ In agreement with this, proteins known to be involved in mitochondria motility along microtubules such as dyneins, dynactins, and kinesins were preserved (Figure 1A and Table S1). We also revealed proteins composing extracellular vesicles in decell muscles (Figure 1A and Table S1). Interestingly, the identification of proteins involved in the ECM remodeling or composing extracellular vesicles strengthen the concept that decell muscle retains biological cues typical of the native tissue.

To determine the tissue‐specificity of protein content preserved in decell scaffolds, we compared our identified proteins to the curated protein composition of different tissues (Figure S1C,D). When we selected the proteins specific for each tissue, skeletal muscle was prevalent with approximately 10‐fold increase in the number of proteins among the tissues under consideration (Figure 1B). Moreover, we found that decell scaffolds preserved specific ECM proteins known to play a role in nerve regeneration and neurite outgrowth, as collagens (I, IV, VI), laminins (α2‐, α4‐, β1‐, and γ1‐chains), and fibronectin.^15^, ^17^, ^42^ These proteins are known to be directly interacting with each other and form a well‐connected network (Figure 1C). Moreover, ECM components of nerves, such as myelin constituents (including myelin basic protein, Mbp, myelin P2 protein, Pmp2, and myelin proteolipid protein, Plp1), and specific ECM proteins of the synaptic basal lamina of neuromuscular junctions (including nidogen‐2, Nid2; laminin α4‐, α5‐ and α2‐chains) were also identified (Table S1 and S2). Altogether, these data support the hypothesis that decellularized muscles retain both muscular and neuronal tissue‐specific ECM components, including proteins that have been shown to drive nerve regeneration and promote neurite outgrowth.

To test whether decell muscles could have an intrinsic neurotrophic effect, scaffolds were cocultured with oSpCs in a 3D environment. The use of this culture system had the aim to retain 3D organization, multiple cell composition, and cell‐ECM interaction of neural cells within organotypic spinal cords,^43^, ^44^ as well as the specific skeletal muscle environment provided by the decell scaffolds. This strategy should allow to better mimic the in vivo innervation process, excluding the contribution of muscle regenerative and systemic responses (ie, inflammation) to the innervation process. In particular, we investigated the ability of decell scaffolds to sustain neural projection sprouting within its 3D environment by culturing oSpC in close proximity to the scaffolds or to attract neural axons when cocultured at a distance from each other.

**FIGURE 2:**
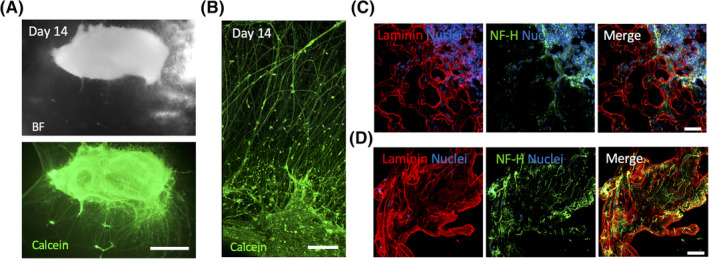
3D culture of oSpC sections. A, Representative stereomicroscope live imaging of Calcein (green) incorporation from oSpC cultured into Matrigel at 14 days after seeding. Scale bar = 1 mm. BF, bright field. B, Two‐photon live imaging of oSpC‐derived neural projection incorporating Calcein (green) at 14 days after seeding into Matrigel. Scale bar = 200 μm. C, *z*‐stack images showing immunostaining for neurofilament‐H (NF‐H, green) and laminin (red) of cross‐sections performed in the middle region of the oSpC at 7 days after seeding into Matrigel. Nuclei were stained with Hoechst (blue). Scale bars = 50 μm. D, *z*‐stack images showing immunostaining for neurofilament‐H (NF‐H, green) and laminin (red) of cross‐sections performed in in the distal region of the oSpC at 7 days after seeding into Matrigel. Nuclei were stained with Hoechst (blue). Scale bars = 50 μm

To reach this aim, we first characterized 3D oSpC culture within Matrigel droplet in absence of the decell scaffolds. Fetal rat SpC were isolated and sectioned in three segments to approximately subdivide the cervical, thoracic, and lumbar/sacral regions. Each single section was then embedded in a Matrigel droplet and cultured up to 14 days. Incremental formation of cellular projections was observed in oSpC cultured in 3D from 2 to 14 days after seeding (Figures 2A and S2A). No differences in culture morphology were observed among oSpC derived from different anatomical regions (data not shown). Calcein uptake in the cellular bodies and projections of the 3D oSpC cultures confirmed the viability of the cells for 14 days (Figures 2A and S2). Interestingly, cellular projections were able to sprout from the SpC body in the 3D environment generated by the ECM constituting the Matrigel droplet (Figure 2B and Movie S1). Immunofluorescence analysis for neuron‐specific class III beta‐tubulin (Tuj1), neurofilament (NF‐H), Choline Acetyltransferase (ChAT), and neuronal nuclei (NeuN) confirmed the maintenance of cholinergic neurons over the cell culture and the axonal identity of the projections identified within the Matrigel (Figures 2C,D, S2C, and S3). The presence of Glial fibrillary acidic protein, localization of which was mainly restricted at the border of the oSpC body, demonstrated the maintenance of multiple cell types during the 3D culture (Figure S3B). Importantly, almost all the nuclei were identified in the body of the oSpC, with the presence of a few sparse cells located distantly from the oSpC body in the Matrigel droplet matrix (Figure S3).

**FIGURE 3:**
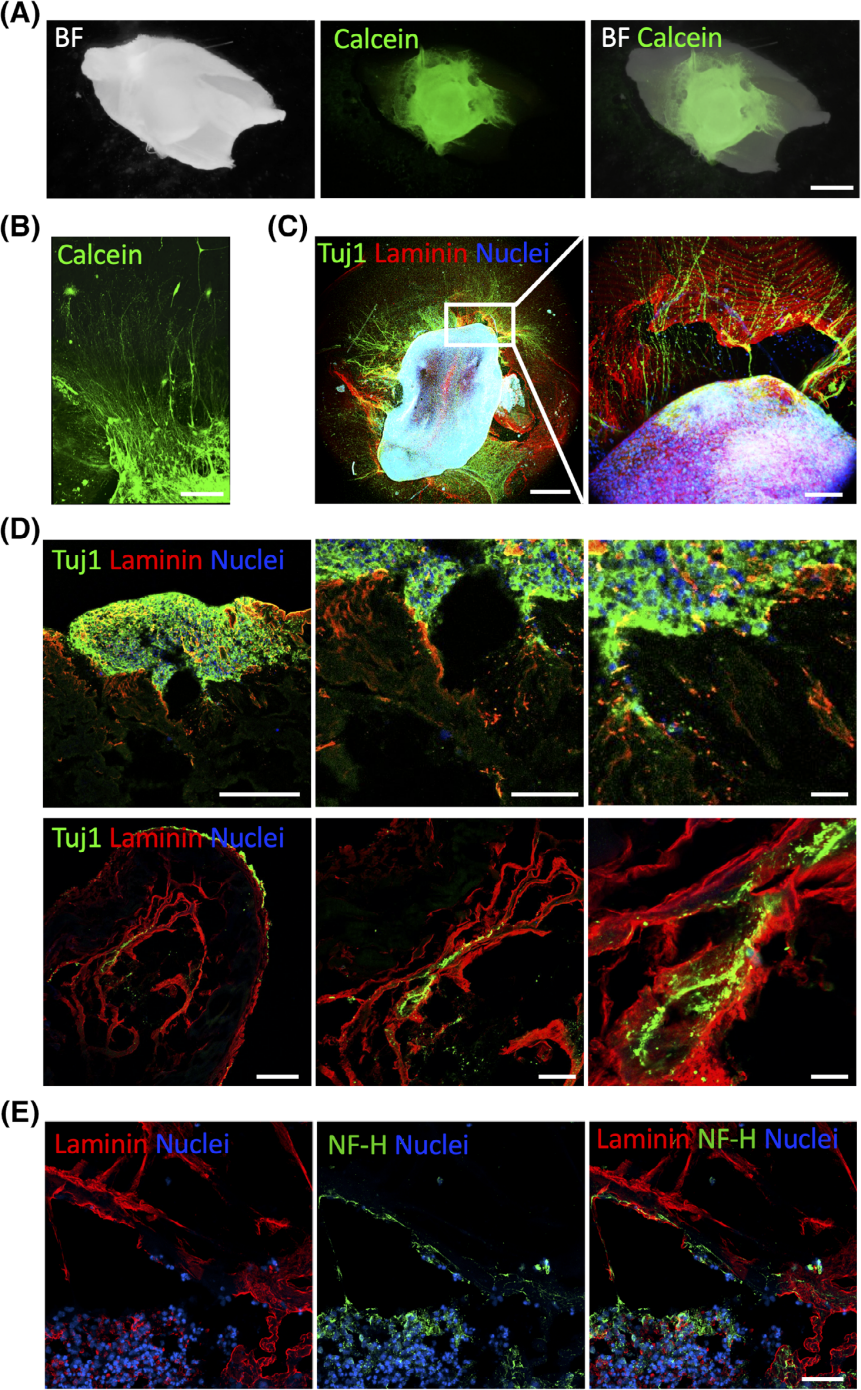
3D coculture of oSpC onto decellularized muscles. A, Representative stereomicroscope live imaging of Calcein (green) incorporation from oSpC cultured onto decellularized muscle at 14 days after seeding. Scale bar = 1 mm. BF, bright field. B, Two‐photon live imaging of SC‐derived neural projection incorporating Calcein (green) at 14 days after seeding onto decellularized muscles. Scale bar = 200 μm. C, *z*‐stack two‐photon image of whole mount oSpC cultured onto decellularized muscle immunostained for Tuj1 (green) and laminin (red) at 14 days after seeding. The inset (right panel) shows high magnification imaging. Nuclei were stained with Hoechst (blue). Scale bar = 500 μm or 200 μm (inset). D, *z*‐stack images showing immunostaining for Tuj1 (green) and laminin (red) of cross‐sections performed in the middle region of the oSpC/decell muscle (upper panels) and in the distal region of the oSpC/decell muscle (lower panels) at 14 days after seeding. Nuclei were stained with Hoechst (blue). Scale bars = 200 μm (left panel), 100 μm (middle panel), and 20 μm (right panel). E, *z*‐stack images showing immunostaining for neurofilament‐H (NF‐H, green) and laminin (red) of cross‐sections performed in the middle region of the oSpC/decell muscle at 14 days after seeding. Nuclei were stained with Hoechst (blue). Scale bars = 50 μm

**FIGURE 4:**
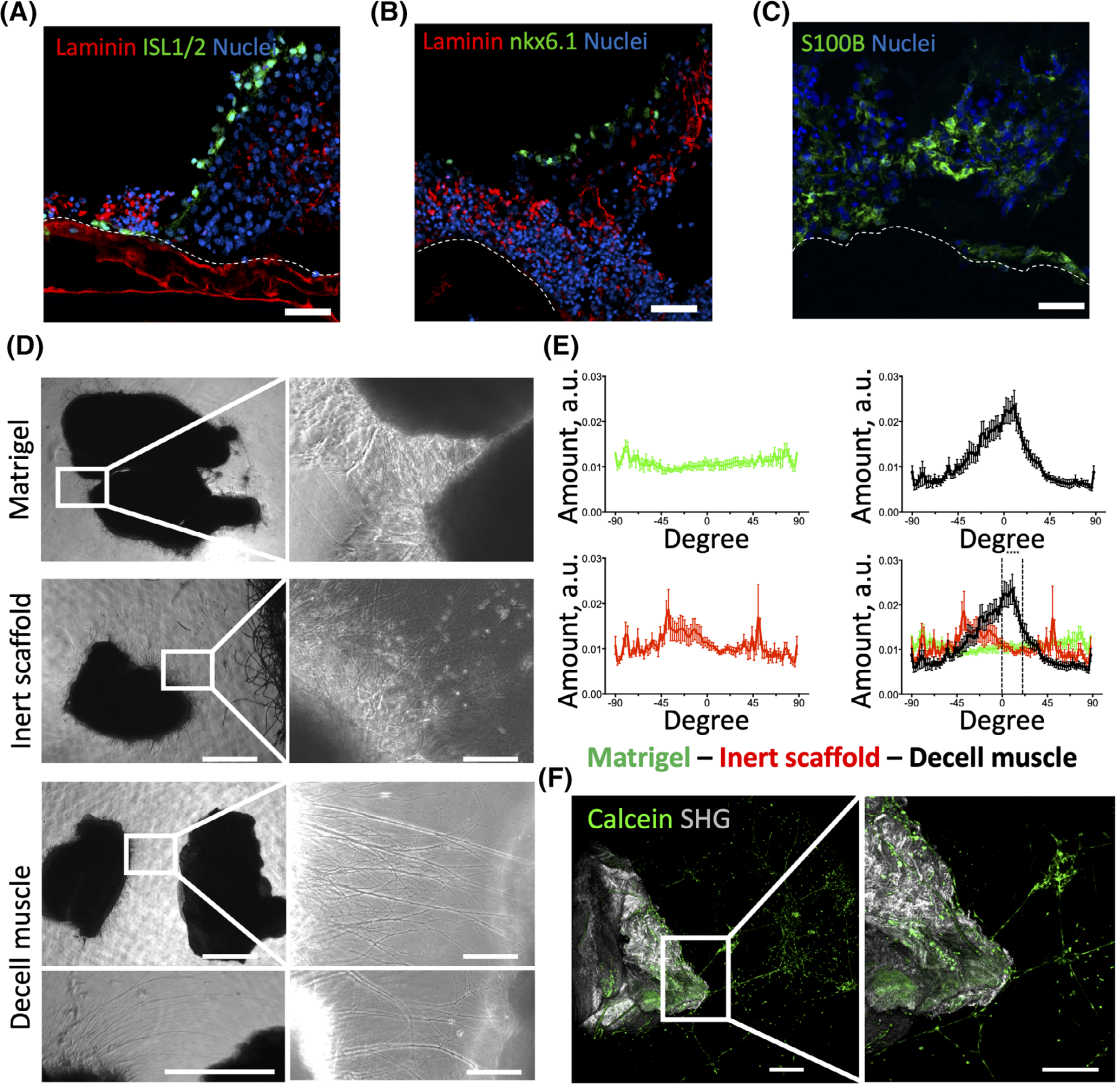
Evaluation of multicellular composition and axonal attractant effect of decellularized muscles on oSpC culture. A‐C, *z*‐stack images showing immunostaining for ISL1/2 (green) and laminin (red; A), nkx6.1 (green) and laminin (red; B), or S100B (green; C) of cross‐sections performed in the middle region of the oSpC/decell muscle at 14 days after seeding. Nuclei were stained with Hoechst (blue). Scale bars = 50 μm. Dashed lines indicate the interface between scaffolds and oSpCs. D, Representative bright field images of oSpC cultured into Matrigel, cocultured with inert scaffolds or cocultured with decell muscles at 4 days after seeding. The insets show axon projections within the Matrigel droplet. Scale bars = 1 mm (left panel) and 100 μm (right panel). E, Quantification of neuronal projection directionality in oSpC section cultured in presence of Matrigel (green), of inert scaffold (red) or decell muscle (black) at 4 days after seeding. Data are shown as mean ± SEM of four independent replicates; multiple comparison one‐way ANOVA (analysis of variance) was used. ***P < .01 among all the experimental groups. F, Two‐photon live imaging of oSpC‐derived neural projection incorporating Calcein (green) that run within the Matrigel toward decell muscle identified with SHG (gray) at 14 days after seeding. The image shows neural projections sprouted from the central body of oSpC, which instead is out from the optical field. Scale bar = 200 μm. SHG, second‐harmonic generation

Next, the oSpC was positioned in close proximity of the decell muscles, that is, onto the scaffolds. First, we used the Calcein incorporation assay to evaluate the oSpC viability upon culture on top of the decellularized scaffolds. Notably, 3D oSpC were vital after 14 days of culture, showing axons that run from the central body of the oSpC over the decell muscles (Figure 3A,B and Movie S2). Also, in the presence of decell muscles, the majority of the cell bodies remained localized within the oSpC and only a few cells were found in a distant position over the scaffolds during the culture (Figure 3). To confirm the neuronal nature of such cellular projections, oSpC cultured onto decell scaffolds were analyzed in whole‐mount via immunostaining (Figure 3C). Consistently with what observed in the absence of decellularized muscles, two‐photon imaging confirmed that neurons within oSpC extend their projections over the decell muscle. The surface of the decell muscles was identified by staining for laminin (ECM component of the muscle basal lamina). To better investigate whether such axons were able to grow within the scaffold, samples were cryo‐sectioned and subjected to immunofluorescence. Analysis performed at different regions of the coculture (middle or distal in respect to the oSpC body) demonstrated that axons were not only confined to the surface of decell muscles, but were also able to invade the ECM, penetrating within the scaffolds and localizing in close proximity to laminin (Figure 3D,E). Further immunofluorescence analysis for the members of the LIM homeodomain (LIM‐HD) transcription factor family Isl1 and Isl2, the homeobox protein Nkx6.1, and S100B confirmed that oSpC cultured onto decell scaffolds preserve a multicellular composition (Figure 4A‐C). Altogether these data demonstrated the ability of decell scaffolds to sustain neural survival and to allow axonal sprouting within their 3D environment.

When used in vivo to repair a resected muscle, decell scaffolds were permissive to innervation along the entire length of the implants. Importantly, this included the median region located far from the host tissue.^11^ Therefore, we also hypothesized that the scaffolds could possess direct neuroattractant properties. To investigate this, oSpC were cocultured with decell muscles by seeding them at a distance from each other and embedding them in Matrigel droplets to allow a gel‐mediated physical connection (Figure 2A). Axonal spouting was monitored during the culture period at 4, 7, and 14 days after seeding (Figures 4D‐F and S5). We first quantified axon directionality, comparing oSpC culture performed in Matrigel droplets (a) in the absence of decellularized muscle, (b) in the presence of decellularized scaffolds, or (c) in the presence of inert scaffold. Four days after seeding, no preferential directions of the axons were observed in oSpC cultured within Matrigel or cocultured with inert scaffolds (Figure 4D,E). Conversely, marked orientation of axons was revealed in oSpC cocultured with decell muscles, with projections sprouting toward the decell muscles (Figure 4D,E). Based on these results, we also evaluated axon length 4 days after seeding. The presence of decell scaffolds did not influence significantly the length of neural projections, when compared to oSpC cultured in Matrigel or in the presence of inert scaffold (Figure S4). Moreover, the preferential organization of neural projections observed during the first days of culture was not appreciated anymore at longer time points, due to incremental sprouting in all the directions (Figure S5). Notably, projections of viable cells were present within the decell scaffold at 14 days after seeding, while nuclei were almost completely retained in the body of the oSpC. This was confirmed via two‐photon microscopy imaging of Calcein incorporating oSpC‐derived projections and by second‐harmonic generation imaging that revealed decell ECM (Figure 4F). These results indicate that decell scaffolds promote neural attraction during the early stages of the 3D coculture. Accordingly, projections sprout toward the scaffold also when oSpC and decell muscles are positioned at a distance from each other.

## 4 DISCUSSION

The perfusion process of decellularization allows for the maintenance of the native skeletal muscle complexity,^7^, ^45^ not only composed of myofibers, but also constituted by other structures such as peripheral nerves. Here, we show that upon decellularization, the skeletal muscle scaffold proteomic composition is much more complex than a mere network of structural ECM proteins associated with the myofibers.

Organotypic cultures of neural slides have been shown to represent a middle ground between dissociated cells and in vivo studies, and have been instrumental in enhancing our understanding of axon guidance.^43^, ^44^ The use of our 3D coculture system allowed to retain cell‐cell and cell‐matrix interactions and to investigate the direct neuro‐attractant effects of decell muscles, excluding other players that could instead operate in vivo during the regeneration of the implants. In particular, our model sustained the preservation of neurons expressing Isl1/2 and Nkx6.1, which are both markers required for motor neuron specification,^46^, ^47^, ^48^ as well as neuronal cells expressing S100B, which is localized in many neural cell‐types, including astrocytes.^49^

It is known that the ECM can either promote or inhibit the elongation of neurites and modulate axon growth.^16^, ^17^, ^18^, ^19^, ^20^ For example, laminin is an adhesive component of the ECM secreted by Schwann cells. Laminin‐rich basal lamina scaffolds have shown pro‐regenerative capability following nerve injury, promoting axonal outgrowth in vitro^15^, ^16^. Moreover, studies aiming at using decell tissues to repair peripheral nerve injuries suggested that the basal lamina of implanted decell scaffolds (that includes collagen type IV, fibronectin, and laminins) could mimic the endoneurial tube, thus promoting in vivo nerve outgrowth and nerve regeneration.^27^ Our proteomic analysis of decell muscles confirmed the preservation of ECM proteins involved in axon growth (such as collagens, laminins, and fibronectin), as well as components of the peripheral nerves such as myelin constituents. Therefore, we can speculate that the observed ability of axons to sprout over and within the scaffolds could be the result of a physical guidance exerted by the structural and molecular composition of the ECM preserved in decell muscle.

Furthermore, the ability of decell muscles to attract neuronal axons strongly supports the hypothesis that the scaffolds have an intrinsic neurotrophic nature. The identification of the mechanism underpinning axon attraction from decell muscles remains an intriguing aspect that needs further investigation. However, the preservation of the signaling components in decell muscles strongly suggests that the scaffold could serve as a *reservoir* of neuro‐attractant molecules. Indeed, decell muscles preserved not only structural proteins of the ECM, but also extracellular vesicles and proteins involved in the ECM remodeling. Extracellular vesicles comprise a heterogeneous population of membrane vesicles with particular lipid, protein, and nucleic acid composition that are considered as an additional mechanism for intercellular communication, including the regulation of signal transduction and cell adhesion.^50^ Interestingly, together with the presence of such signaling components, we also demonstrated that decell scaffolds preserve chemokines, such as vascular endothelial growth factor (VEGF) and insulin‐like growth factor (IGF‐1), abundance of which was estimated to be approximately 39 pg/mg wet decell tissue and 44 pg/mg wet decell tissue, respectively.^11^ Both IGF‐1 and VEGF are neurotrophic factors that have been shown to promote peripheral nerve regeneration, axonal targeting and outgrowth, and to be protective in both in vitro and in vivo models of neuronal degeneration.^51^, ^52^, ^53^ A number of studies have demonstrated that soluble factors released from the ECM and its degradation products themselves are capable of recruiting both neural differentiated cells and progenitors to the site of remodeling,^54^ as well as Schwann cells from products derived from decellularized small intestinal submucosa.^55^ It is, therefore, not unconceivable that signaling molecules could be released from the decell muscles during the time in culture, creating the chemotactic gradient, within the 3D culture system that promoted axonal sprouting toward the scaffolds.

## 5 CONCLUSION

Our study demonstrated that decell muscles obtained preserving the native tissue environment have direct neurotrophic properties. This strongly suggests that our model could represent a powerful tool to investigate in vitro axon sprouting and guidance within a complex native‐like skeletal muscle 3D environment. Finally, the increasing understanding of the neurotrophic properties of decell scaffolds can open new perspectives for tissue engineering approaches aimed at promoting in vivo reinnervation and functional skeletal muscle regeneration.

## CONFLICT OF INTEREST

The authors declared no potential conflicts of interest.

## AUTHOR CONTRIBUTIONS

P.R., V.S.: collection and assembly of data, data analysis and interpretation; M.F.M.G., S.P., P.D.C.: performed skeletal muscle decellularization; M.C., N.E., C.L.: performed proteomic data analysis and interpretation; P.C.: isolated fetal rat spinal cords; A.U.: conception and design, financial support, data analysis and interpretation, manuscript writing.

## Supporting information


**Supplementary Figure S1** **Proteomic analysis.**
**A.** Venn diagram of the reviewed reference proteomes of *Homo sapiens*, *Mus musculus* and *Rattus norvegicus*, according to Uniprot database. **B.** Cumulative distribution function of the number of detected unique peptides/protein. **C.** Number of proteins identified in our data that are annotated to be expressed at the protein level in the indicated tissues. **D.** Number of proteins that are annotated to the indicated number of tissues. **C‐D.** Reference data of human proteins per tissue are described in Methods section.

**Supplementary Figure S2** **Establishment and characterization of oSpC 3D culture into Matrigel.**
**A.** Representative bright field images of oSpC sections 3D cultured into Matrigel at 2, 7 and 14 days after seeding. Scale bars, 1 mm (upper panel) and 100 μm (lower panel). **B.** Representative stereomicroscope live imaging of Calcein (green) incorporation from oSpC 3D culture at 7 days after seeding. Scale bar, 1 mm; BF, bright field. **C.** Immunofluorescence staining for Tuj1 (green) of whole mount oSpC at 7 days after. Nuclei were stained with Hoechst (blue). Scale bar 1 mm; inset scale bar 200 μm.

**Supplementary Figure S3** **Characterization of oSpC 3D culture 14 days after seeding into Matrigel.**
**A.** Representative Z‐stack image of oSpC longitudinal‐sections immunostained for ChAT (green) and NeuN (red). Lower panels show high magnification images. Nuclei were stained with Hoechst (blue). Scale bars, 500 μm (upper panel) and 200 μm (lower panel). **B**. Representative Z‐stack image of oSpC longitudinal‐sections immunostained for Tuj1 (green), GFAP (red) and laminin (gray). Lower panels show high magnification images. Nuclei were stained with Hoechst (blue). Scale bars, 100 μm. **C**. Z‐stack images showing immunostaining for neurofilament‐H (NF‐H, green) and laminin (red) of cross‐sections performed in the distal region of the oSpC at 14 days after seeding. Nuclei were stained with Hoechst (blue). Scale bars, 50 μm.

**Supplementary Figure S4** **Evaluation of axonal length of oSpC culture 4 days after culture.**
Quantification of neuronal projection length in oSpC cultured in presence of Matrigel (green), of inert scaffold (red) or decell muscle (black) at 4 days from seeding. Data are shown as mean ± s.d. of 4 independent replicates; one‐way ANOVA with Tukey's multiple comparison test were used; n.s. not statistically significant.

**Supplementary Figure S5** **Evaluation of axonal attractant effect of decellularized muscles on oSpC culture.**
**A.** Representative bright field images of oSpC cultured into Matrigel, co‐cultured with inert scaffolds or co‐cultured with decell muscles at 7 and 14 days after seeding. Scale bars, 1 mm (left panel) and 100 μm (right panel). **B**. Quantification of neuronal projection directionality in oSpC section cultured in presence of Matrigel (green), of inert scaffold (red) or decell muscle (black) at 7 and 14 days from seeding. Data are shown as mean ± s.e.m. of 4 independent replicates; multiple comparison one‐way ANOVA was used.

**Supplementary Table 1** List of all the 2081 detected proteins in this study.

**Supplementary Table 2** Classification of all the 2081 detected proteins in this study according to their localization.

**Supplementary Movie 1** 3D reconstruction of SC‐derived neural projection incorporating Calcein (green) 14 days after seeding into Matrigel. Scale bars are included in the red grid that shows 3D orientation.

**Supplementary Movie 2** 3D reconstruction of SC‐derived neural projection incorporating Calcein (green) 14 days after seeding onto decell muscles. Scale bars are included in the red grid that shows 3D orientation.

## Data Availability

All data are available in the main text or the Supporting Information and tables.
