# Supplementary figures and images for: Decellularized skeletal muscles display neurotrophic effects in three‐dimensional organotypic cultures
Source: Stem Cells Transl Med. 2020 Jun 24;9(10):1233–43. doi: 10.1002/sctm.20-0090 (PMC7519766; doi:10.1002/sctm.20-0090)

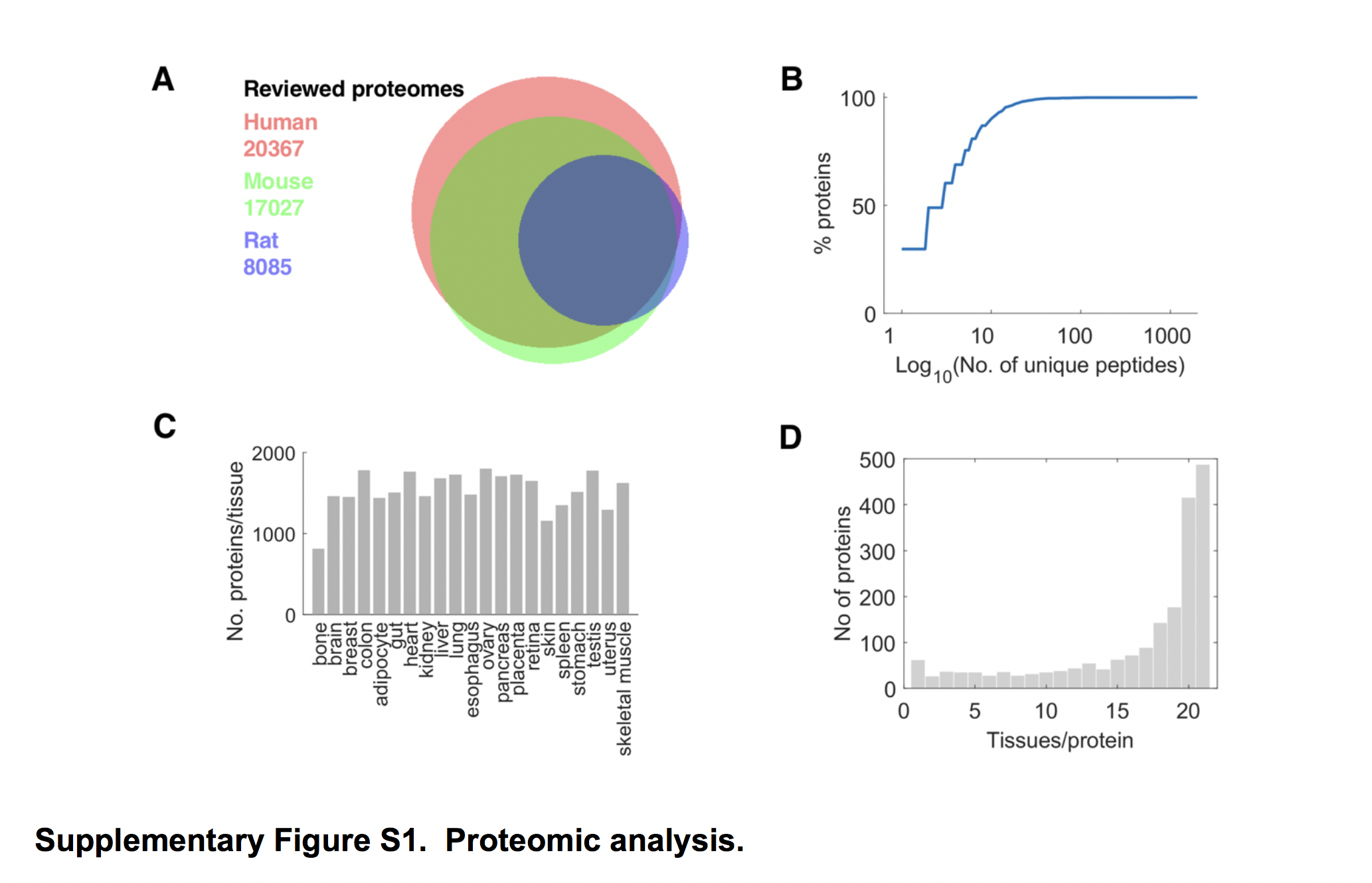

Supplement: Supplementary file 1 — Supplementary Figure S1 Proteomic analysis. A. Venn diagram of the reviewed reference proteomes of Homo sapiens, Mus musculus and Rattus norvegicus, according to Uniprot database. B. Cumulative distribution function of the number of detected unique peptides/protein. C. Number of proteins identified in our data that are annotated to be expressed at the protein level in the indicated tissues. D. Number of proteins that are annotated to the indicated number of tissues. C‐D. Reference data of human proteins per tissue are described in Methods section. [file SCT3-9-1233-s001.tiff]

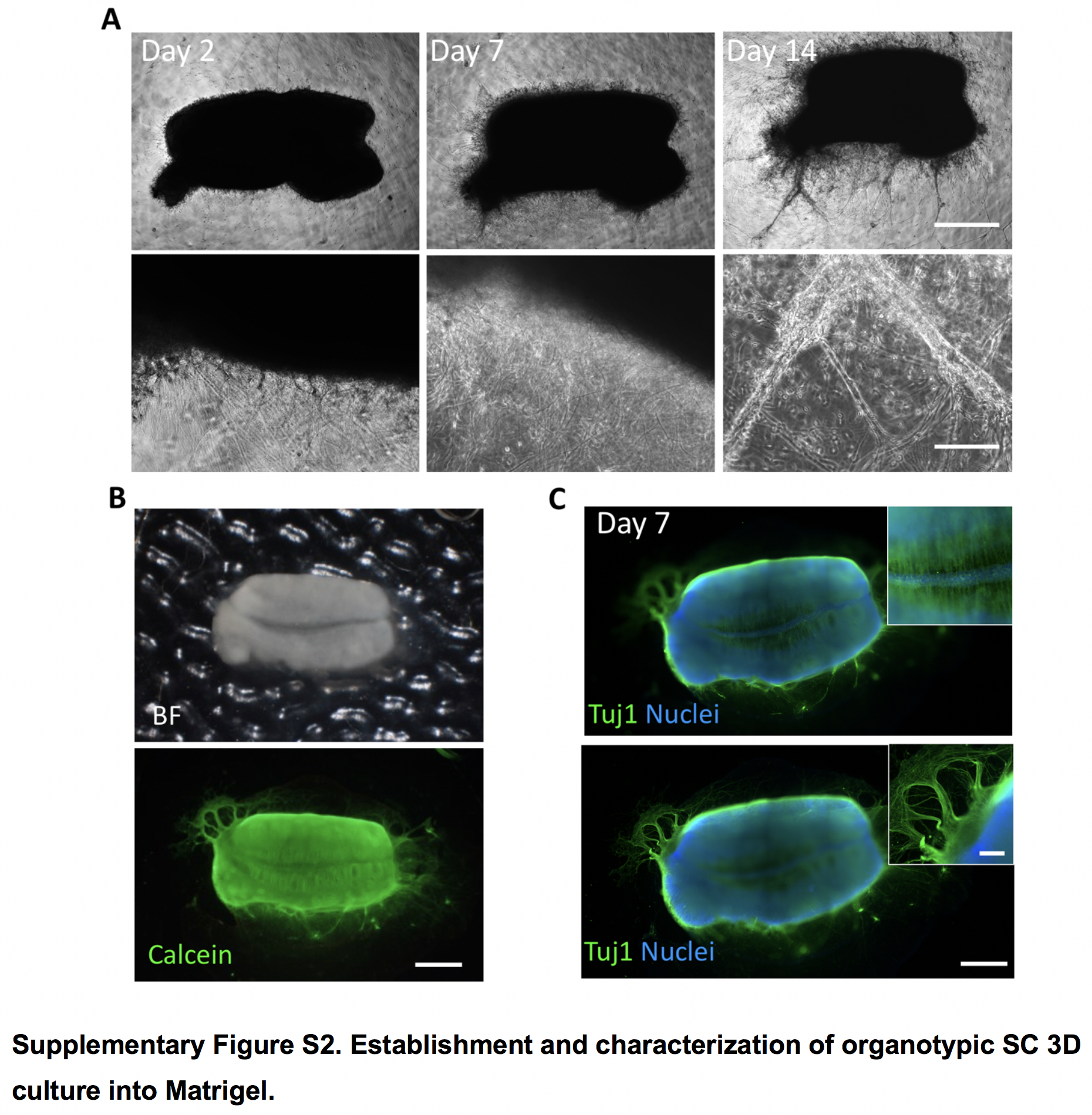

Supplement: Supplementary file 2 — Supplementary Figure S2 Establishment and characterization of oSpC 3D culture into Matrigel. A. Representative bright field images of oSpC sections 3D cultured into Matrigel at 2, 7 and 14 days after seeding. Scale bars, 1 mm (upper panel) and 100 μm (lower panel). B. Representative stereomicroscope live imaging of Calcein (green) incorporation from oSpC 3D culture at 7 days after seeding. Scale bar, 1 mm; BF, bright field. C. Immunofluorescence staining for Tuj1 (green) of whole mount oSpC at 7 days after. Nuclei were stained with Hoechst (blue). Scale bar 1 mm; inset scale bar 200 μm. [file SCT3-9-1233-s004.tiff]

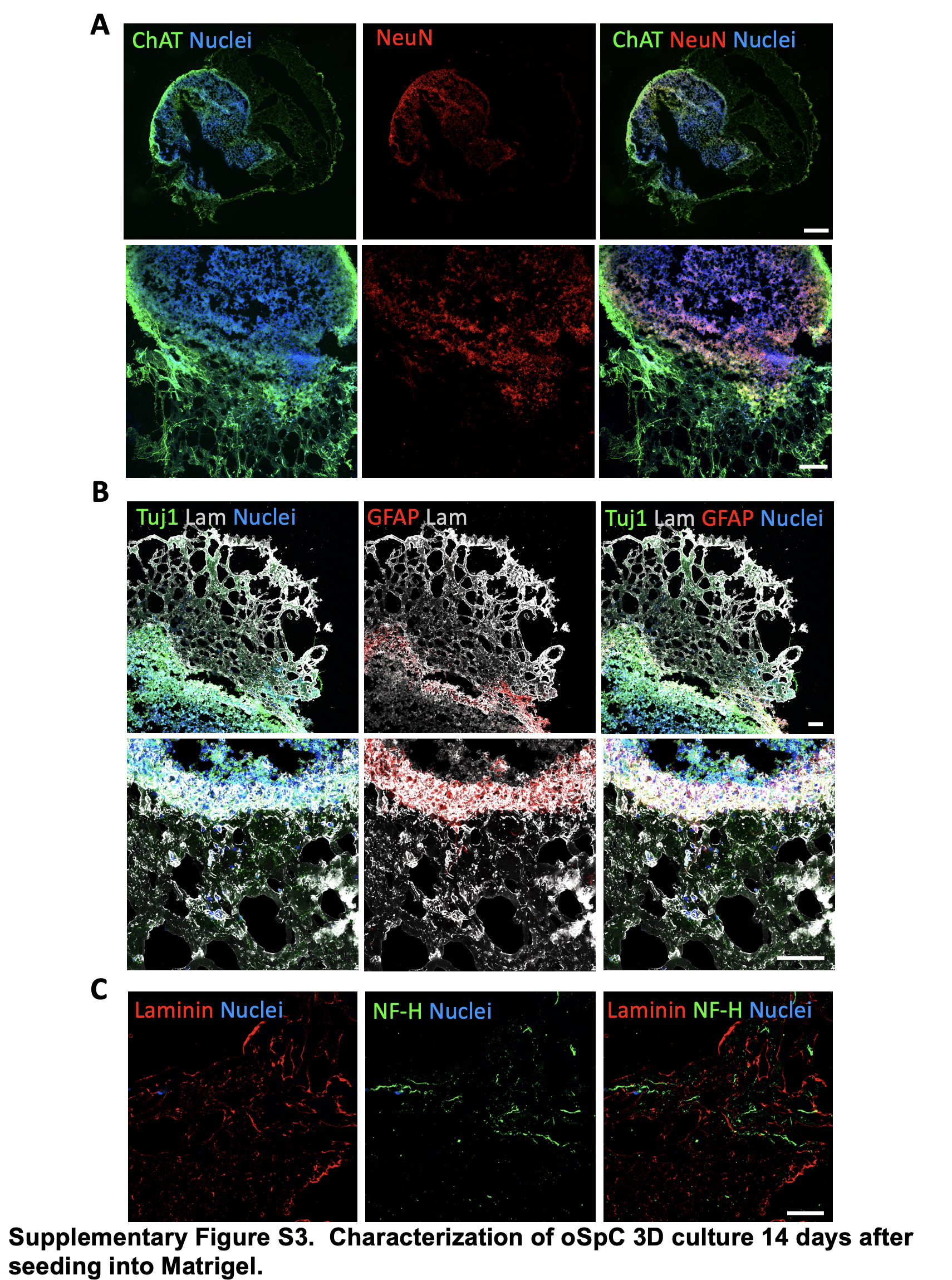

Supplement: Supplementary file 3 — Supplementary Figure S3 Characterization of oSpC 3D culture 14 days after seeding into Matrigel. A. Representative Z‐stack image of oSpC longitudinal‐sections immunostained for ChAT (green) and NeuN (red). Lower panels show high magnification images. Nuclei were stained with Hoechst (blue). Scale bars, 500 μm (upper panel) and 200 μm (lower panel). B. Representative Z‐stack image of oSpC longitudinal‐sections immunostained for Tuj1 (green), GFAP (red) and laminin (gray). Lower panels show high magnification images. Nuclei were stained with Hoechst (blue). Scale bars, 100 μm. C. Z‐stack images showing immunostaining for neurofilament‐H (NF‐H, green) and laminin (red) of cross‐sections performed in the distal region of the oSpC at 14 days after seeding. Nuclei were stained with Hoechst (blue). Scale bars, 50 μm. [file SCT3-9-1233-s007.tiff]

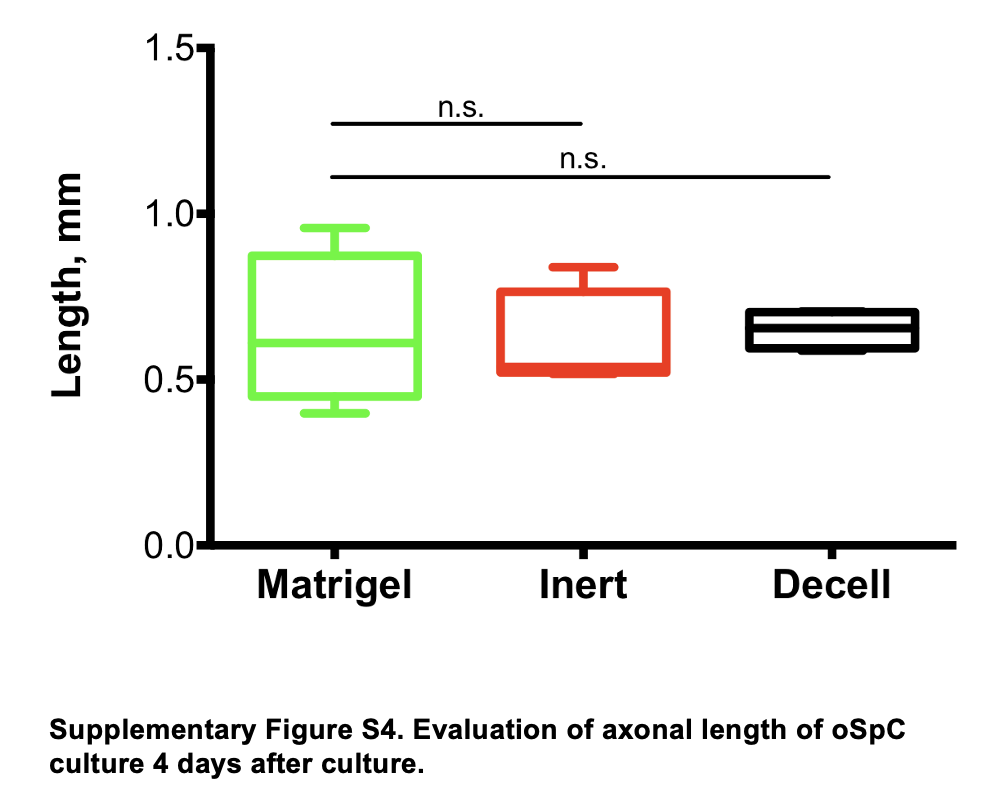

Supplement: Supplementary file 4 — Supplementary Figure S4 Evaluation of axonal length of oSpC culture 4 days after culture. Quantification of neuronal projection length in oSpC cultured in presence of Matrigel (green), of inert scaffold (red) or decell muscle (black) at 4 days from seeding. Data are shown as mean ± s.d. of 4 independent replicates; one‐way ANOVA with Tukey's multiple comparison test were used; n.s. not statistically significant. [file SCT3-9-1233-s008.tiff]

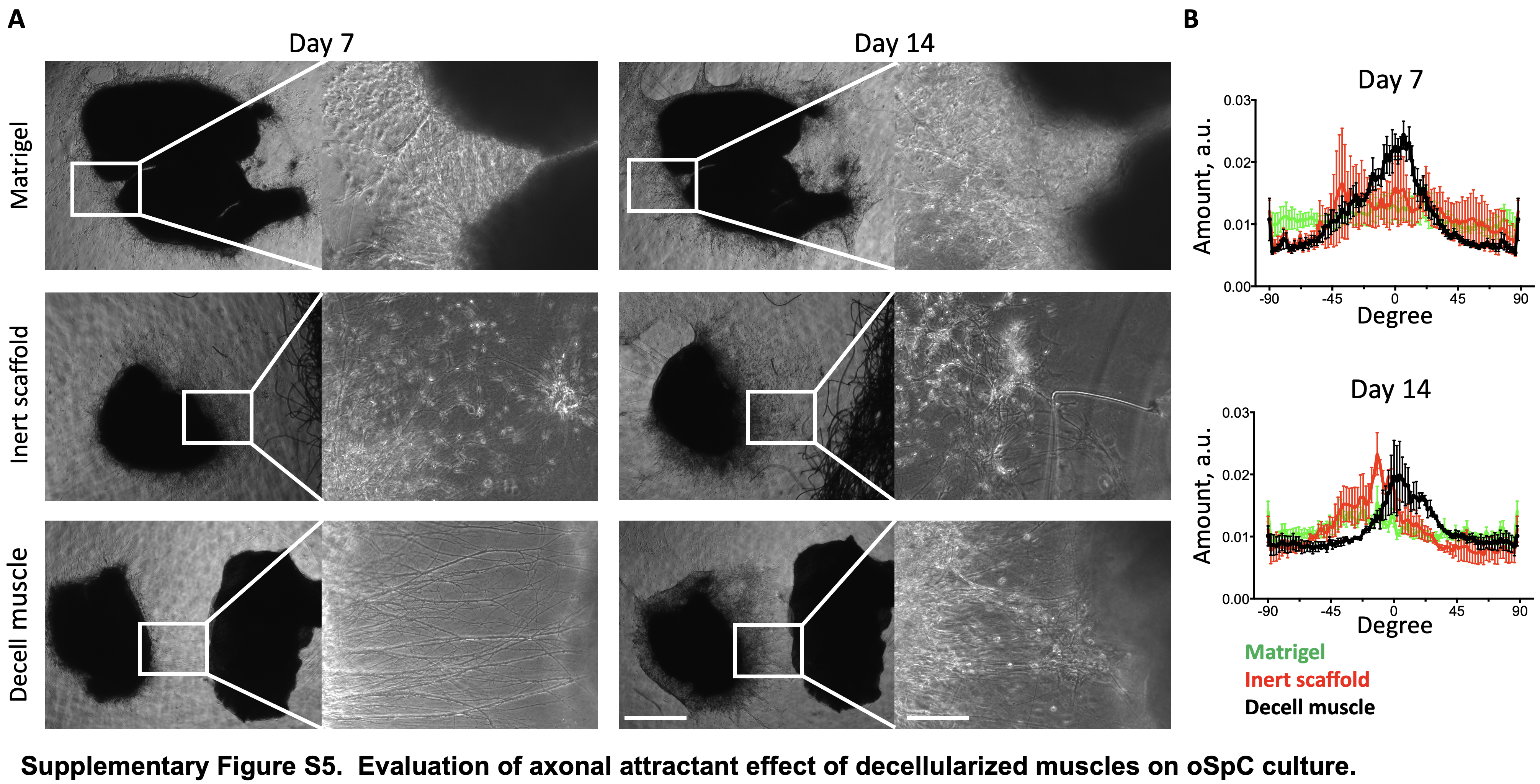

Supplement: Supplementary file 5 — Supplementary Figure S5 Evaluation of axonal attractant effect of decellularized muscles on oSpC culture. A. Representative bright field images of oSpC cultured into Matrigel, co‐cultured with inert scaffolds or co‐cultured with decell muscles at 7 and 14 days after seeding. Scale bars, 1 mm (left panel) and 100 μm (right panel). B. Quantification of neuronal projection directionality in oSpC section cultured in presence of Matrigel (green), of inert scaffold (red) or decell muscle (black) at 7 and 14 days from seeding. Data are shown as mean ± s.e.m. of 4 independent replicates; multiple comparison one‐way ANOVA was used. [file SCT3-9-1233-s009.tiff]

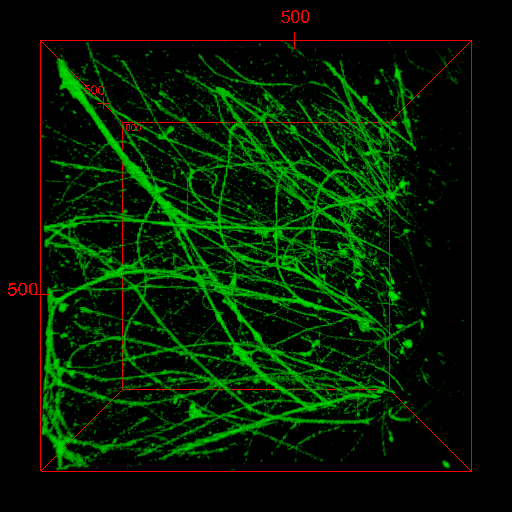

Supplement: Supplementary file 8 — Supplementary Movie 1 3D reconstruction of SC‐derived neural projection incorporating Calcein (green) 14 days after seeding into Matrigel. Scale bars are included in the red grid that shows 3D orientation. [file SCT3-9-1233-s002.gif]

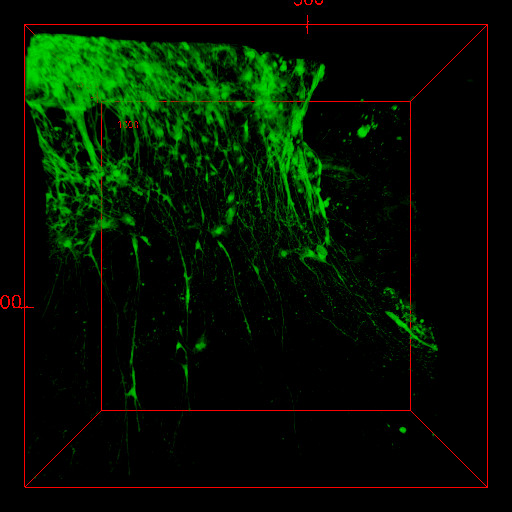

Supplement: Supplementary file 9 — Supplementary Movie 2 3D reconstruction of SC‐derived neural projection incorporating Calcein (green) 14 days after seeding onto decell muscles. Scale bars are included in the red grid that shows 3D orientation. [file SCT3-9-1233-s005.gif]
